# Analyses of blood-derived host biomarkers for pulmonary tuberculosis diagnosis in human immunodeficiency virus co-infected individuals in sub-Saharan Africa: a systematic review and meta-analysis

**DOI:** 10.3389/ftubr.2024.1377540

**Published:** 2025-01-08

**Authors:** Antony M. Rapulana, Thabo Mpotje, Nondumiso Mthiyane, Theresa K. Smit, Timothy D. McHugh, Mohlopheni J. Marakalala

**Affiliations:** ^1^School of Laboratory Medicine and Medical Science, University of Kwazulu-Natal, Durban, Kwazulu-Natal, South Africa; ^2^Basic and Translational Science, Africa Health Research Institute, Durban, Kwazulu-Natal, South Africa; ^3^Centre for Clinical for Clinical Microbiology, Division of Infection and Immunity, University College London, London, United Kingdom; ^4^Institute for Global Health, University College London, London, United Kingdom

**Keywords:** cytokines, chemokine, systematic review, TB diagnosis, biomarkers, HIV-TB co-infection

## Abstract

**Objective:**

Our objective was to conduct a review of host blood-derived biomarkers as potential diagnostic targets for pulmonary TB and as alternative tests to identify active tuberculosis in HIV co-infected individuals.

**Methods:**

A systematic review and meta-analysis of host blood-derived biomarkers with potential for diagnosis of active tuberculosis in HIV co-infected individuals was conducted. Cochrane Library, Embase, MEDLINE, PubMed and Web of Science databases were searched up to 7 November 2023. A hierarchical summary receiver operating characteristic (HSROC) model was used to evaluate the pooled sensitivity, specificity, positive likelihood ratio (PLR), negative likelihood ratio (NLR) and diagnostic odds ratio (DOR) of the following potential biomarkers: C-reactive protein (CRP), Interferon gamma induced protein-10 (IP-10), Neopterin, IGRA, Kynurenine to tryptophan (K/T) ratio and use of different panels of combined biomarkers; including 5 biomarker panel (IL-6, INF-y, MIG, CRP, and IL-18), 4 biomarker panel (IL-6, IL-21, INF-y, IL-1a), 6 biomarker panel (APO-ACIII, CXCL1, CXCL9, CCL8, CCL-1, and CD56), and 9 biomarker panel (Alpha-2-macroglobulin, fibrinogen, CRP, MMP-a, transthyretin, complement factor H, INF-y, IP-10, and TNF-α).

**Results:**

Twenty-three studies were included. The pooled sensitivity of CRP, IP-10, Neopterin, combined biomarker signatures, IGRA and K/T ratio were 77% (60–88), 79% (72 - 84), 82% (43–96), 78% (64–88), 71% (65–76), 95% (90–98), respectively and the pooled specificity were 90% (80–96), 82% (59–93), 42% (22–66), 85% (73–92), 33% (18–54), and 95% (82–99), respectively.

**Conclusion:**

CRP, IP-10, K/T ratio and the panels of multiple combined biomarkers that include the following cytokines, chemokines, and acute phase proteins IL-6, INF-y, MIG, CRP, IL-18, IL-21, IL-1a, APO-ACIII, CXCL1, CXCL9, CCL8, CCL-1, CD56, Alpha-2-macroglobulin, fibrinogen, MMP-a, transthyretin, complement factor H, IP-10, and TNF-α are potential blood biomarkers that can aid TB diagnosis in HIV co-infected individuals.

## Introduction

The diagnosis of pulmonary tuberculosis (TB) in resource limited areas is mainly by chest X-ray, Acid Fast Bacilli (AFB) staining and molecular methods such as GeneXpert (1). The use of GeneXpert MTB/RIF as a first-line diagnostic test for TB is recommended by WHO (2); however the test has not been fully implemented across all sub-Saharan African countries (3). These countries carry a severe burden of TB infection which is aggravated by emergence of drug resistant strains, a high prevalence of HIV co-infection, poverty, and people living in remote areas without proper access to health facilities (4). In response to the burden of disease, the “End TB strategy” targets a 90% reduction in patients suffering from TB, and a 95% reduction in deaths from TB by 2035 (5). However, studies have shown that deaths among HIV-infected individuals as a result of co-infection with TB are significantly higher in sub-Saharan African low- and middle-income countries (LMIC) compared to high-income countries (HIC) (6, 7).

During TB infection, *Mycobacterium tuberculosis* (*Mtb*) is engulfed by innate immune cells including macrophages, which recruit other immune cells such as T cells and B cells to form a granuloma that contains *Mtb* (8). Cytokines and chemokines play a role in this recruitment of immune cells to the site of mycobacteria infection (9) and so they have been proposed as blood-derived biomarkers with potential for the diagnosis of TB (10–12). However, the clinical value of blood biomarkers remains to be evaluated in a setting in sub-Saharan Africa with a high prevalence of both tuberculosis and HIV.

Diagnosis of TB in HIV-positive individuals can be complex: (I) HIV infection can modify the clinical presentation of TB, leading to atypical or less specific symptoms, (II) HIV-positive individuals often have coinfections or comorbidities that can complicate the clinical picture and may mimic TB symptoms, (III) HIV-induced immunosuppression can lead to atypical immune responses to TB, affecting the performance of conventional TB diagnostic tests such as tuberculin skin tests and interferon-gamma assays (13–16). Two meta-analyses reported the potential of IP-10 and CRP for diagnosis of TB from predominantly HIV negative studies (17, 18). By comparison, this systematic review and meta-analysis focuses on the evaluation of chemokines, cytokines, and acute phase protein as diagnostic biomarkers of TB in HIV co-infected individuals in a sub-Saharan African setting with a high prevalence of both TB and HIV.

## Methods

This review was developed in accordance with the Preferred Reporting for Systematic Review and Meta-Analysis protocols (PRISMA-P) diagnostic test accuracy criteria and was registered by the international database of prospectively registered systematic reviews in health and social care (PROSPERO) (CRD42021277685). Ethical approval was not required for this study.

### Literature search

The search strategy was designed by combining the medical (MeSH) terms: “‘tuberculosis,' ‘latent tuberculosis,' ‘LTBI,' ‘TB,' ‘human Immunodeficiency syndrome,' ‘HIV,' ‘Sub-Saharan Africa,' ‘Biomarkers,' ‘assay,' ‘assays,' ‘bio-signature,' ‘bio-signatures,' ‘expression,' ‘marker,' ‘markers,' ‘profile,' ‘profiling,' ‘profiles,' ‘signature,' ‘signatures,' ‘surrogate endpoint,' ‘test,' ‘tests,' ‘tool,' ‘tools.”' in the following electronic databases: Embase, PubMed, and Web of Science up to and including 7 November 2023. The MeSH terms were used together using “OR,” and the results were further combined using “AND” to obtain the result. The full search strategy is described in the Supplementary data 2.

### Literature selection

We included studies that assessed the diagnostic accuracy of blood-based biomarkers for TB in HIV co-infected individuals, TB negative in HIV positive individuals, in sub-Saharan Africa. Only manuscripts written in English were included. Studies focused only on human pulmonary TB. For inclusion, there must have been a confirmed TB disease by either culture (liquid or solid), Acid fast bacilli (AFB) or GeneXpert MIT/RIF, as well as confirmed HIV infection. The studies that met our inclusion criteria were published between 2009 and 2023. We included studies that reported sensitivity, specificity, or sufficient information on any biomarker/s of pulmonary TB diagnosis assessed to construct tables of outcome. We included studies focused on both hospital and community-based participants of all ages. We also included case control, cohort, cross sectional studies and only studies that have clear reference standards for laboratory diagnosis of pulmonary TB. Reviews, letters, case reports, clinical trials, conference abstracts, and animal experiments were excluded.

### Data extraction

Two reviewers (AR and NM) independently performed quality assessment on the extracted data and any discrepancies were resolved by discussion and consensus. The following data were extracted: author, year, country where study was conducted, participant information, reference standards, tests index, cut-offs, true positive (TP), false positive (FP), false negative (FN) and true negative (TN).

### Quality assessment

The quality assessment of diagnostic accuracy studies tool-2 (QUADAS-2) was used to evaluate the risk of bias and applicability in each study (19). Each reviewer performed an independent quality assessment of included studies by evaluating four domains (patient selection, index tests, reference standard, and flow and timing) for risk bias and three domains (patient selection, index tests and reference standard) for applicability. The Review Manager software (version 5.4, Cochrane Collaboration) was used to process the quality assessment of the included studies.

The first domain is patient selection i.e., selection of the participants based on a consecutive or at random basis, case-control design was avoided, and verifying whether the study avoided unnecessary exclusions. The participants of articles included in this review were also required to have the test condition. Thus, the risk of bias is high since only participants suspected of TB were selected. The second domain is the index test i.e., index test results interpreted without knowledge of the results of the reference standard and accurate explanation of detection threshold. The third domain is the reference standard 99% accuracy, but interpretation without considering the results of the index test and ensuring that all patients were assessed using the same reference standard. The last or fourth domain is the flow and timing (for risk bias only) describing the patients receiving the index test, the time interval between index tests, and reference standard. After independent evaluations, the reviewers discussed the article. Each domain was discussed to achieve a single view.

### Statistical analysis

We used sensitivity and specificity of the biomarker reported in the studies to recalculate the sample numbers in each group. R software (version 4.12) was used to perform the statistical analyses. The pooled sensitivity, specificity, diagnostic odd ratios (DOR) were calculated, and summary receiver characteristics curves (SROC) were plotted for diagnostic efficiency of each biomarker of TB in HIV infected individuals from relevant articles relating to each biomarker. Index Q^*^ was calculated from the corresponding value of DOR using Walter's formula (20). The DOR reflects the effectiveness of the index tests: DOR > 1 indicates that positive tests suggest active TB and DOR < 1 indicates that negative tests suggest that disease is absent. *I*^2^ statistic was used to quantify the amount of variation across studies. A *p*-value of < 0.05 indicated the presence of heterogeneity among included studies.

## Results

### Literature search

**Figure 1:**
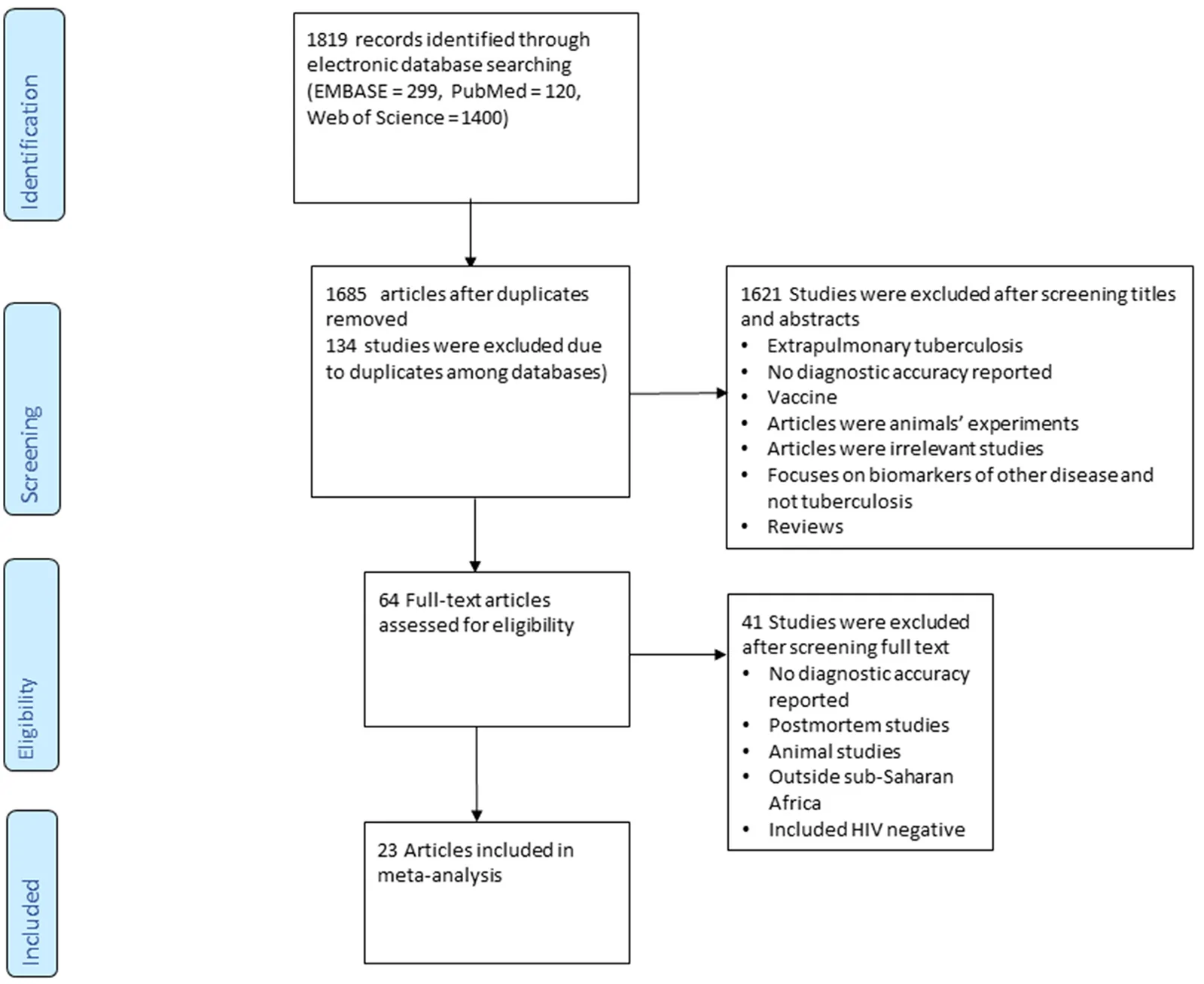
PRISMA flow diagram of inclusion and exclusion criteria.

A total of 1,819 articles were identified from database searches (Embase = 299, PubMed = 120 and Web of Science = 1,400). Sixty-four papers were included and of the 1,755 excluded studies, 134 were duplicates; 1,621 studies were excluded after screening titles and abstracts relating to any of the following: (extrapulmonary tuberculosis, no diagnostic accuracy reported, vaccine studies, animal experiments, focus on biomarkers of other diseases, and reviews), 2 were reviews. Forty-one studies were excluded after full paper screening if they (Included HIV negative only, animal studies/experiments, conducted outside Sub-Saharan Africa, or Postmortem studies). Ultimately 23 studies were eligible for inclusion in this study (Figure 1).

The characteristics of the 23 eligible studies are shown Table 1 (21–45). All eligible studies were published in English between 2009 and 2023. Three (13%) studies included only children (21, 28, 31), seventeen (74%) studies included only adults (23, 26–30, 33, 46), three (13%) studies did not report the ages (24, 25).

**Table 1:** Main characteristics of included studies.

**References**	**Country study conducted**	**Samples**	**Method**	**Reference standard**	**Index test (s)**	**Test and cutoffs**	**TP**	**FP**	**FN**	**TN**
Aabye et al. (35)	Tanzania	Plasma	QFT-IT ELISA	Sputum Culture	QFT-IT	0.35 IU/mL	44	24	NR	NR
Adu-Gyamfi et al. (36)	South Africa	Plasma	LC-MS	Culture, Chest radiography and Clinical signs and syptoms	Kyn to Trp Ratio	0.8	36	1	1	69
Adu-Gyamfi et al. (37)	South Africa	Plasma	ELISA	Liquid culture, flouresence microscopy and Expert MTB/RIF	Kyn to Trp Ratio	0.1	68	4	105	12
Alvarez et al. (21)	South Africa	NR	NR	Culture	CRP	>11 mg/dl	82	6	4	136
Ashenafi et al. (38)	Ethiopia	PBMC	ELISA and Flow cytometry	Culture	ALS	NR	29	47	NR	NR
Bedell et al. (34)	Malawi	blood	CRP latex	Culture and MTB/RIF GeneXpert	CRP	>10 mg/L	45	45	7	151
Cattamanchi et al. (40)	Uganda	Peripheral blood and bronchoalveolar lavage	ELISPOT	Culture	IGRA	NR	26	10	46	12
Cattamanchi et al. (41)	Uganda	Peripheral blood	ELISPOT	Culture	IGRA	NR	93	33	59	51
Chegou et al. (22)	South Africa	Quantiferon Supernatant	Luminex multiplex	MGIT and Chest radiography	IP-10	>6,768 pg/mL	12	25	7	32
Ciccacci et al. (23)	Mozambique	Plasma	ELISA	MTB/RIF	CRP	>10 mg/L 10 nmol/L	16	6	5	116
				GeneXpert	Neopterin		49	143	3	53
Drain et al. (24)	South Africa	Blood	Dimension RXL analyser	AFB and Culture (Solid and Liquid)	CRP	>8 mg/L	43	24	2	24
Farr et al. (25)	Uganda	Plasma	Multiplex	AFB, MGIT and MTB/RIF GeneXpert	Combination of CRP with IL-6, INF-y, MIG	20, 23, 2994, 248, 69 mg/L	44	5	10	15
Gersh et al. (26)	Kenya	Blood and spot	NR	AFB, MGIT and MTB/RIF GeneXpert	CRP	>10 mg/L	1	38	5	340
Lawn et al. (27)	South Africa	Serum	ELISA	Liquid culture, fluorescence microscopy and MTB/RIF GeneXpert	CRP	< 10 mg/L	69	179	12	239
Kisuya et al. (42)	Kenya	PBMC Supernatant	Flow Cytometry and ELISA	Culture	IFN-y, TNF-a, IL-2, and IL-12	NR	36	4	5	41
Lundtoft et al. (28)	Ghana	Quantiferon Spernatant	Luminex multiplex	Chest radiography and sputum culture	Combination of IL-6, IL-21, TNF-α and IL-1α	NR	18	7	NR	NR
Manngo et al. (29)	South Africa	QuantiFERON Supernatant	Luminex Multiplex	MTB/RIF GeneXpert and MGIT	Combination of Apo-ACIII, CXCL1, CXCL9, CCL8, CCL-1, CD56	< 20,360.2, >381.7, >940.3, −0.1554, < 124,132 ng/mL	26	9	9	60
Morris et al. (30)	South Africa and Malawi	Serum	Luminex multiplex	Culture	CRP	NR	110	37	12	90
					P-10		99	22	23	105
					Nine Protein signature		112	37	10	90
Petrone et al. (31)	Uganda	Blood	Luminex	TB culture (Liquid and solid)	IP-10	209.1 pg/mL	25	5	7	74
Rajan et al. (43)	Uganda	blood	PCR	Culture	5-transcript signature		38	2	10	30
Ruperez et al. (44)	Zambia and South Africa	blood	Alere Afinion AS100 analyser	Culture and MTB/RIF Expert	CRP	5 mg/L	18	7	133	118
Uwimaana et al. (33)	Uganda	Plasma and serum	ELISA	Sputum culture	Neopterin and HO-1	>10.12 and >8.95 ng/mL	40	28	30	42
Yoon et al. (45)	Uganda	whole blood	POC	Culture and MTB/RIF Expert	CRP	10 mg/L	145	18	283	731

AFB, acid-fast bacillus; CRP, C-reactive protein; ELISA, enzyme-linked immunosorbent assay; IP-10, induced protein 10; MGIT, mycobacteria growth indicator tube; TB, tuberculosis; TP, true positive; FP, false positive; FN, false negative; TN, true negative; Nine protein signature (fibrinogen, alpha-2-macroglobulin, CRP, MMP-9, transthyretin, complement factor H, IFN-gamma, IP-10, and TNF-alpha); NR, not reported.

The reference standard for pulmonary TB diagnosis includes the use of MTB/RIF GeneXpert, Acid Fast Bacillus (AFB) staining, culture (solid and liquid culture) and fluorescence microscopy. Study design, samples used, method, reference standard, index test, cut-off, true positive, false positive, false negative, and true negative for each study are summarized in Table 1. Five individual biomarkers CRP, IGRA, IP-10, Kynurenines to tryptophan ratio and neopterin were reported frequently. Five studies reported mixed biomarker sets for diagnosis of tuberculosis.

**Figure 2:**
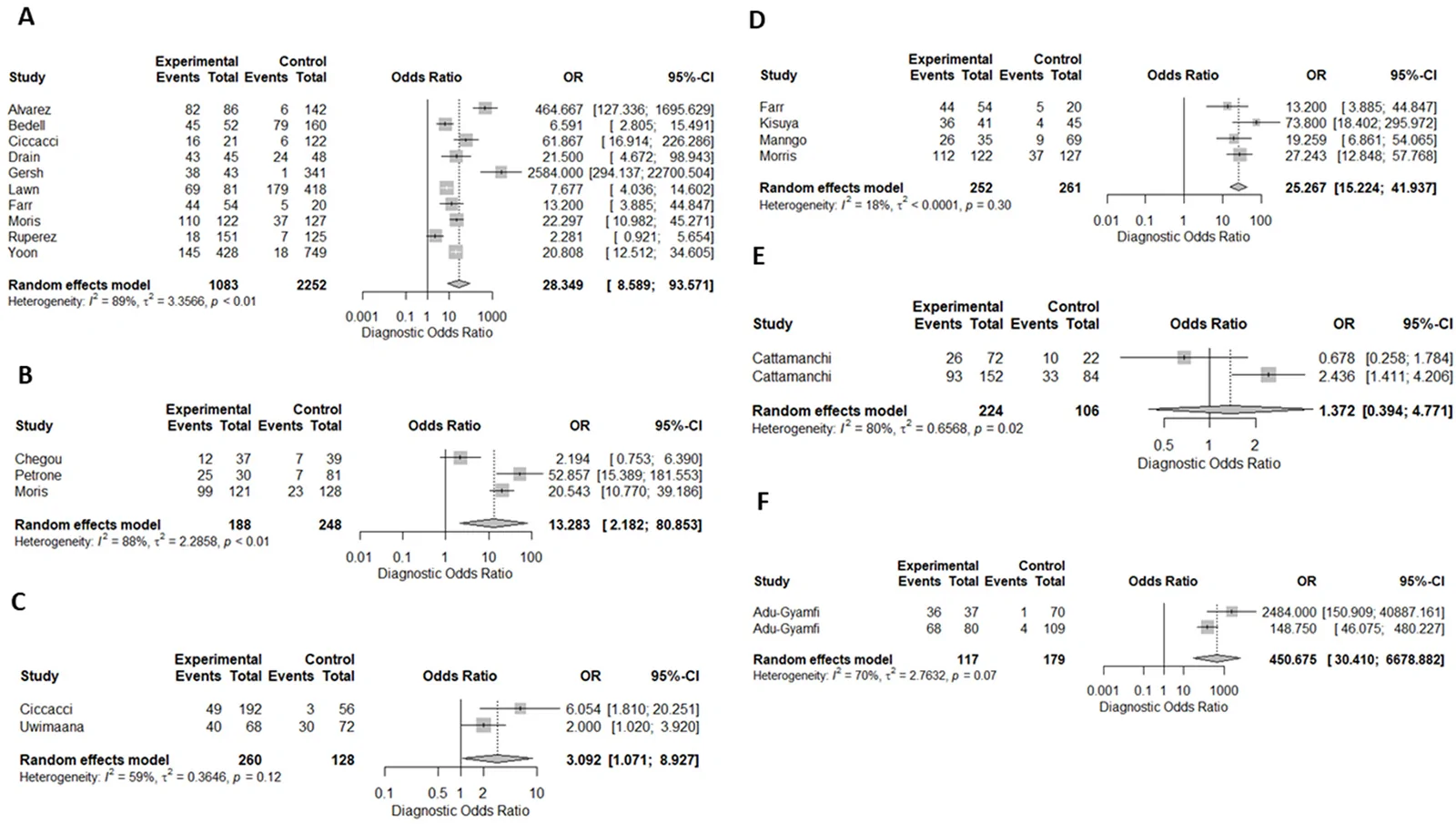
Forest plots of diagnostic odds ratio for **(A)** CRP, **(B)** IP-10, **(C)** Neopterin, **(D)** combination of biomarkers, **(E)** IGRA, and Kynurenines to tryptophan ratio in the diagnosis of TB, **(F)** K/T ratio in the dioagnosis of TB. OR, odds ratio; CI, confidence interval.

**Figure 3:**
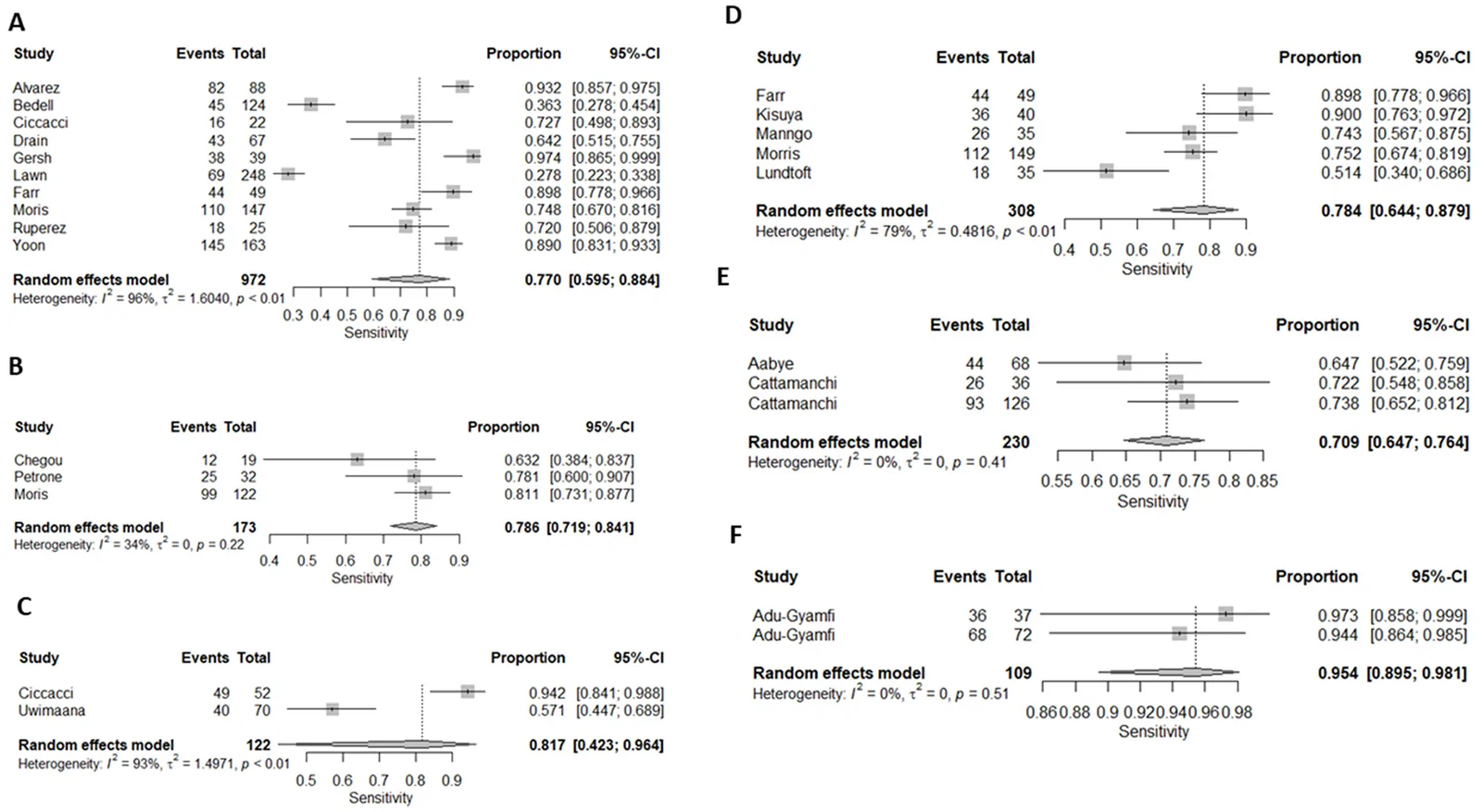
Forest plots of **(A)** CRP's, **(B)** IP-10, **(C)** Neopterin, **(D)** combination of biomarkers, **(E)** IGRA, and Kynurenines to tryptophan ratio sensitivity for diagnosis of active tuberculosis, **(F)** K/T ratio sensitivity for diagnosis of active tuberculosis.

**Figure 4:**
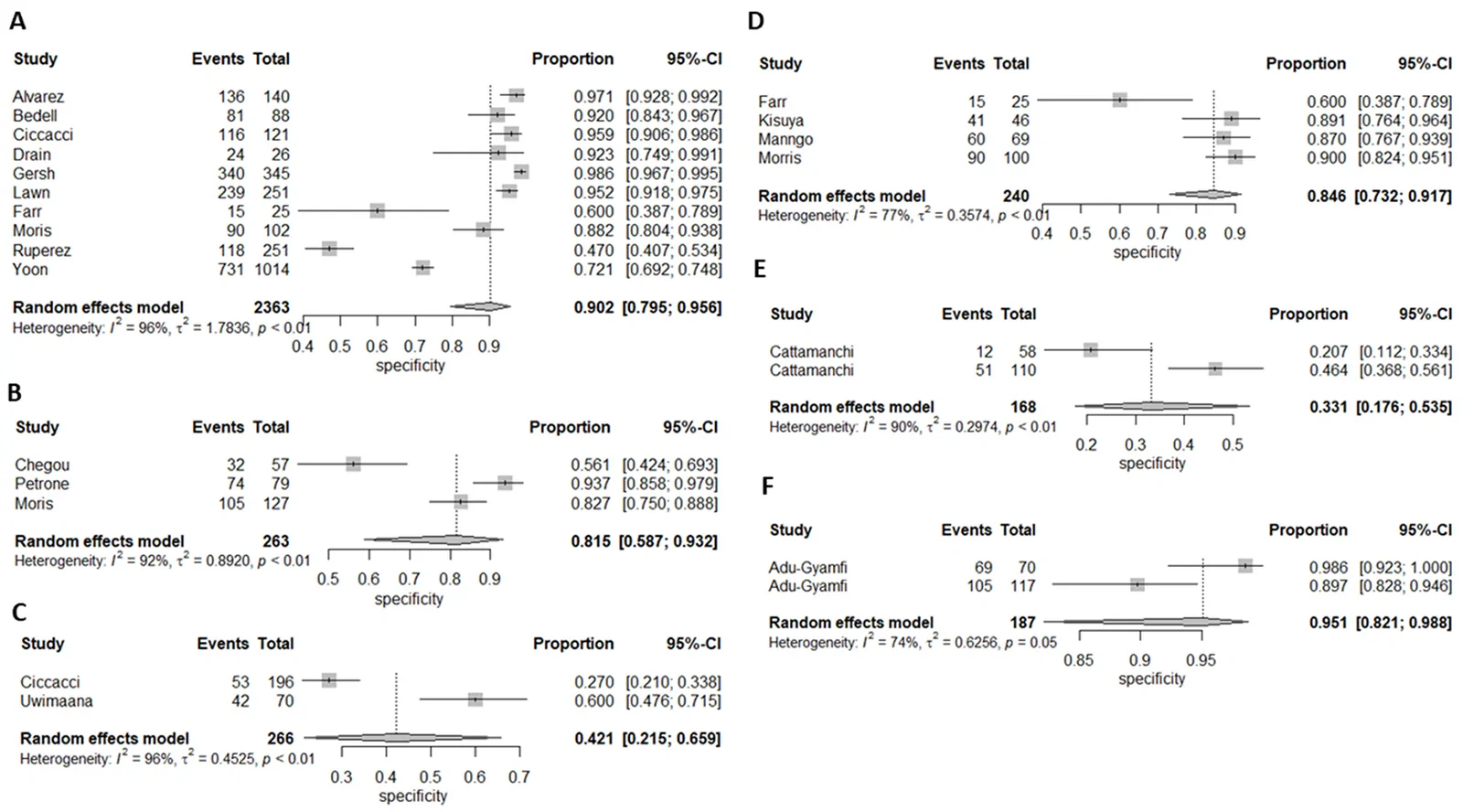
Forest plot of **(A)** CRP's, **(B)** IP-10, **(C)** Neopterin, **(D)** combination of biomarkers, **(E)** IGRA, and Kynurenines to tryptophan ratio for specificity for diagnosis of active tuberculosis, **(F)** K/T ratio sensitivity for diagnosis of active tuberculosis.

Ten (43%) studies used CRP as the index test (21, 23–27, 30, 34, 44, 45, 47) and a total of 1,882 participants were included. The diagnostic odds ratio (DOR) was 28.349 (8.589–93.571) (Figure 2). The pooled sensitivity was 77% (59.5–88.4) and pooled specificity 90.2% (79.5–95.6) (Figures 3, 4). The heterogeneity for CRP was significant with *I*^2^ of 96% for sensitivity and 96% for specificity with both *p*-value of less than < 0.01, respectively.

Three (13%) studies used IGRA (35, 40, 41) as index test, with a total of 398 participants. The pooled sensitivity was 70.9% (64.7–76.4), pooled specificity was 33.1% (17.6–53.5), and DOR was 1.372 (0.394–4.771) (Figures 3, 4). The heterogeneity analysis of IGRA yielded an *I*^2^ of 0% for sensitivity and significant heterogeneity of pooled specificity with *I*^2^ of 90% with *p*- value of less than < 0.01.

The three (13%) studies which determined the diagnostic accuracy of IP-10 (22, 30, 31), included a total of 436 participants. The pooled sensitivity was 79% (72–84), pooled specificity was 82% (59–93), and DOR was 13.28 (2.18–80.85) (Figures 3, 4). The heterogeneity analysis of IP-10 yielded an *I*^2^ of 34% for sensitivity and significant heterogeneity of pooled specificity with *I*^2^ of 88% with *p*- value of < 0.01.

Two (8.7%) studies used K/T ratio (36, 37) as an index test, with a total of 296 participants. The pooled sensitivity was 95.4% (89.5–98.1), pooled specificity was 95.1% (82.1–98.8), and DOR was 450.675 (30.410–6678.882) (Figures 3, 4). The heterogeneity analysis of K/T ratio yielded an *I*^2^ of 0% for sensitivity and significant heterogeneity of pooled specificity with *I*^2^ of 74% with p- value of < 0.05.

Two (8.7%) studies used neopterin (23, 33) as an index test, with a total of 388 participants. The pooled sensitivity 82% (42–96), pooled specificity 42% (22–66), and DOR 3.09 (1.07–8.93) (Figures 3, 4). The heterogeneity analyses of neopterin yielded an *I*^2^ of 93% for sensitivity and 96% for specificity and *p*-value of < 0.01 respectively.

One (4.3%) study reported antibodies in lymphocyte supernatant (38) and another study (4.3%) reported at 5-transcript signature (43) for diagnosis of pulmonary TB in HIV co-infected participants. The pooled sensitivity and specificity were not done for ALS and 5-transcript signatures.

Among the included studies, five (21.7%) looked at a combination of biomarkers as signatures to evaluate the diagnostic accuracy of active tuberculosis. The combination of biomarker signatures in the five studies included five biomarkers (IL-6, INF-y, MIG, CRP, and IL-18) (25), four biomarkers (IL-6, IL-21, INF-y, IL-1a) (28), six biomarkers (APO-ACIII, CXCL1, CXCL9, CCL8, CCL-1, and CD56) (29), nine biomarkers (Alpha-2-macroglobulin, fibrinogen, CRP, MMP-a, transthyretin, complement factor H, INF-y, IP-10, and TNF-α) (30), and four biomarkers (INF-y, TNF-a, IL-2, and IL-12) (42). The DOR was 25.267 (15.224–41.937) (Figure 2). The pooled sensitivity and specificity were 78.4% (64.4–87.9) and 84.6% (73.2–91.7) (Figures 3, 4). There was significant heterogeneity in both pooled sensitivity and specificity with *I*^2^ of 79 and 77%, and the *p*-value of < 0.01 and < 0.01, respectively. We performed SROC analysis to assess the power of CRP, IP-10, Neopterin, selected combined biomarkers, IGRA, and K/T ratio and all had good diagnostic accuracy (Supplementary Figure 1).

### Quality assessment

QUADAS-2 was used to assess risk bias and applicability of the study. Two studies (26, 29) showed high risk of bias and 8 (24, 25, 28, 30, 31, 38, 40, 41) had unclear risk for patient selection. Three studies (21, 28, 30) had high risk for index test and two (24, 43) showed an unclear risk of bias for index test. For applicability concerns one study (28) showed high risk of bias and one (29) showed unclear risk of bias (Supplementary Figure 2).

## Discussion

The findings from this systematic review suggest that analyses of CRP (77.0 and 90.0%), IP-10 (78.6 and 81.5%), K/T ratio (95.4 and 95.1%), and a panel of multiple combined cytokines (Table 2), chemokines and acute phase proteins (78.4 and 84.6%) in TB/HIV coinfected individuals are promising potential biomarkers, with pooled sensitivity and specificity of more than 75% in diagnosing TB. With future validation, these blood-based markers may provide alternative ancillary methods for diagnosis of pulmonary TB in HIV co-infected individuals.

**Table 2:** The role/function of cytokines and chemokines and cells producing them.

**Biomarkers**	**Cells producing**	**Function/role**
CRP	Liver, atherosclerotic plaques by activated vascular cells	Sent into bloodstream in response to inflammation
IP-10	T-cells	It is chemokine secreted from cells stimulated with type I and II IFNs and LPS, is a chemoattractant for activated T cells. It plays important role in recruiting activated T cells into sites of tissue inflammation.
Neopterin	Macrophages, lymphocytes, dendritic cells	It is biomarker for immune system activation. The monocytes and dendritic cells activate gene expression of inducible NOS to increase neopterin levels. It is member of the pteridine family, is derived from guanosine triphosphate via guanosine triphosphate cyclohydrolase and is released from macrophages as a consequence of T cell-dependent interactions involving interferon-y
IL-6	Macrophages	It is a soluble mediator with a pleiotropic effect on inflammation, immune response, and hematopoiesis
INF-y	Lymphocytes	It is effector cytokine with pleiotropic role associated with antiproliferative, pro-apoptotic and antitumor mechanisms
MIG	Endothelial cell, infiltrating neutrophil, and macrophage	It is CXC chemokine active as a chemoattractant for activated T cells.
IL-18	Kupffer cells or macrophage	It is proinflammatory cytokine that has pleiotropic function involved in the regulation of both innate and acquired immune responses, playing a key role in autoimmune, inflammatory, and infectious diseases
IL-1a	macrophage or activated monocytes and senescent cells	pro-inflammatory that stimulates the activity of genes involved in inflammation and immunity
IL-21	T helper cells	It is cytokine that has regulatory effects on cells of the immune system, including natural killer (NK) cells and cytotoxic T cells that can destroy virally infected or cancerous cells
APO-ACIII	Liver	
CXCL1/MGSA	Macrophage, Neutrophil, Epithelial cell and TH17 population	It is chemokine that plays a pivotal role in the host immune response by recruiting and activating neutrophils for microbial killing at the tissue site
CXCL9	Monocytes, Endothelial cells, Fibroblasts and cancer cells	It is chemokine which plays role to induce chemotaxis, promote differentiation and multiplication of leukocytes, and cause tissue extravasation
CCL8/MCP2	T helper type 2 cells	It is belonging to CC chemokine sub family that plays a pivotal role in the control of leukocyte chemotaxis, HIV enry and other inflammatory disease
CCL-1	Activated monocytes, Macrophage, T lymphocytes and Endothelial cells	It is glycoprotein that binds to the chemokine receptor CCR8 and induces Ca2+ influx, chemotaxis and regulate apoptosis
CD56/NCAM	Natural Killer cells	The recognition of target cells and in the induction of cytotoxicity
Alpha-2-macroglobulin	liver, macrophages, fibroblasts and adrenocortical	Binding host or foreign peptides and particles, thereby serving as humoral defense barriers against pathogens in the plasma and tissue
Fibrinogen	liver hepatocyte	Formation of fibrin that binds together platelets and some plasma proteins in a hemostatic plug
Transthyretin	liver, pancreatic cells, retina, epithelial cells of choroid plexus	Is a transport protein in the plasma and cerebrospinal fluid that transports the thyroid hormone thyroxine and retinol to the liver
Complement factor H	Podocytes	It is a soluble complement regulator essential for controlling the alternative pathway in blood and on cell surfaces
TNF-α	Activated macrophages, T lymphocytes, natural killer cells	Inflammatory cytokine that is responsible for a diverse range of signaling events within cells, leading to necrosis or apoptosis

Blood-based biomarkers could play a crucial role in improving TB diagnosis in HIV positive individuals by providing a less invasive, more accessible, and faster diagnostic method. It has been shown that individuals with HIV-TB co-infection may have a lower bacterial load in the sputum, therefore making traditional diagnostic tool like sputum microscopy less effective (48, 49). It has been shown that in TB infection, myeloid cells, including macrophages and neutrophils, are essential in the immune response mounted to Mycobacterium tuberculosis acting as the first line of defense by phagocytosing and attempting to kill the bacteria (8, 50). Myeloid-derived suppressor cells (MDSCs) can also amass during TB infection, potentially suppressing immune responses and contributing to disease advancement (51). In HIV infection, myeloid cells such as macrophages and dendritic cells are important targets for and may serve as reservoirs of HIV, enabling the virus to endure even during antiretroviral therapy, as well as facilitate the spread to CD4+T cells (52). In the case of HIV and TB coinfection, HIV infection may impair the function of myeloid cells, reducing their ability to control *M.tb* which may lead to a higher risk of latent TB reactivation and more advanced disease (53). In addition, the dysfunction of macrophages and dendritic cells due to HIV can exacerbate TB outcome. Most of the studied biomarkers with potential to diagnose TB in HIV-co-infected individuals are produced by myeloid cells including macrophages and neutrophils, as well as dendritic cells (54–57). Thus, mechanistically, these myeloid specific responses would be expected to be higher in TB-HIV co-infection compared to HIV infection alone, and could aid in the diagnoses of TB in people living with HIV. However, such proposed mechanism need validation.

The results of this study were consistent with those reported in the systematic review by Santos et al. where both high- and low-income countries were included (18), however our study considered HIV infected individuals only. The meta-analysis by Santos et al. reported higher sensitivity of IP-10, the same sensitivity of CRP and low specificity of both IP-10 and CRP in HIV uninfected participants compared to our study and showed that IP-10 and CRP can also be used to diagnose pulmonary TB in patients with other lung diseases (18). It should be noted that some studies tested different sets of biomarkers, the SROC plots include diagnostic accuracy of combined cytokines, chemokines, and acute phase proteins from those studies.

It has been estimated that the current limitation in TB diagnosis has led to ~3.6 million TB cases never being detected or properly treated (58). There is an urgent need for more sensitive and specific TB diagnostic tests with a short turnaround time. Even with the extensive rollout by WHO of MTB/RIF GeneXpert, the low sensitivity in sputum samples and challenges of diagnosis in infants and children, who may not be able to provide sputum samples (32), remains a critical limitation on improved diagnostics. The limitation of this study was the lack of data to analyze levels of the biomarkers at different time points of TB disease, from asymptomatic subclinical disease to infection and to severe advanced cavitary diseases. Secondly, we could not analyze the source of significant heterogeneity for CRP, IP-10, and Neopterin studies due to fewer number of studies, However, for CRP, the use of different sample types (blood, plasma, dried blood spots, and serum) may have contributed to the observed heterogeneity. Third limitation of the study is inability to investigate impact of CD4 count on the biomarkers.

In conclusion, the CRP, IP-10, neopterin, K/T ratio and panels of multiple combined biomarkers are promising tests for diagnosis of tuberculosis in HIV co-infected individuals. However, more studies need to be conducted to examine the combination of biomarkers in HIV infected individuals vs. HIV uninfected individuals to support the finding of this systematic review. We recommend future studies are also conducted to determine and evaluate the use of biomarker algorithms to (i) accurately diagnose active TB in HIV coinfected individuals; (ii) assess the impact of HIV treatment on TB diagnosis; (iii) determine the transition from latent to active TB; and (iv) assess if there is any strain variation in diagnosing TB using host biomarkers.

## Data Availability

The original contributions presented in the study are included in the article/Supplementary material, further inquiries can be directed to the corresponding author.
