# Supplementary material for: Analyses of blood-derived host biomarkers for pulmonary tuberculosis diagnosis in human immunodeficiency virus co-infected individuals in sub-Saharan Africa: a systematic review and meta-analysis
Source: Front Tuberc. 2025 Jan 8;2:1377540. doi: 10.3389/ftubr.2024.1377540 (PMC13495002; doi:10.3389/ftubr.2024.1377540)
Supplement: Supplementary file 1 [file Data_Sheet_1.docx]

**Supplemental Figure 1**: The summary receiver operating characteristic (SROC) curve for assessment of A. CRP, B. IP-10, C. Neopterin, D. Combination of biomarkers, E. IGRA, and F. Kynurenines to tryptophan ratio for tuberculosis diagnosis

**D**

**B**

**A**

**C**

**E**

**F**

**Supplemental Figure 2**: A. Showing the 4 domain (patient selection, index test, reference standard, flow, and timing) risk of bias for studies and B. Showing the 4 domain (patient selection, index test and reference standard) applicability of studies
