# Supplementary material for: Analyses of blood-derived host biomarkers for pulmonary tuberculosis diagnosis in human immunodeficiency virus co-infected individuals in sub-Saharan Africa: a systematic review and meta-analysis
Source: Front Tuberc. 2025 Jan 8;2:1377540. doi: 10.3389/ftubr.2024.1377540 (PMC13495002; doi:10.3389/ftubr.2024.1377540)
Supplement: Supplementary file 2 [file Data_Sheet_2.docx]

**Supplemetary data 2**: Showing the search strategy and date used for different database

Web of Science **07 November 2023**

**"Angola" OR "Benin" OR "Botswana" OR "Burkina Faso" OR "Burundi" OR "Cameroon" OR "Cape Verde" OR "Central African Republic" OR "Chad" OR "Comoros" OR "Congo" OR "Cote dIvoire" OR "Djibouti" OR "Equatorial Guinea" OR "Eritrea" OR "Ethiopia" OR "Gabon" OR "Gambia" OR "Ghana" OR "Guinea" OR "Kenya" OR "Lesotho" OR "Liberia" OR "Madagascar" OR "Malawi" OR "Mali" OR "Mauritania" OR "Mauritius" OR "Mozambique" OR "Namibia" OR "Niger" OR "Nigeria" OR "Reunion" OR "Rwanda" OR "Sao Tome" OR "Senegal" OR "Seychelles" OR "Sierra Leone" OR "Somalia" OR "South Africa" OR "Sudan" OR "Swaziland" OR "Tanzania" OR "Togo" OR "Uganda" OR "Western Sahara" OR "Zambia" OR "Zimbabwe" OR "subsahara*" OR "sub saharan africa"** (All Fields) and **"Tuberculosis" OR "TB"** (All Fields) and **"Human Immunodeficiency Virus"OR "HIV"** (All Fields) and **"assay" OR "assays" OR "biomarker" OR "biomarkers" OR "bio-signature" OR "bio-signatures" OR "expression" OR "marker" OR "markers" OR "profile" OR "profiling" OR "profiles" OR "signature" OR "signatures" OR "surrogate endpoint" OR "test" OR "tests" OR "tool" OR "tools"** (All Fields) and **"blood" OR "SERUM" OR "plasma"** (All Fields)

Pubmed **07 November 2023**

("sub saharan africa"[All Fields] OR "africa south of the sahara"[All Fields]) AND ("analysis"[MeSH Subheading] OR "analysis"[All Fields] OR "assay"[All Fields] OR "biological assay"[MeSH Terms] OR ("biological"[All Fields] AND "assay"[All Fields]) OR "biological assay"[All Fields] OR "assay s"[All Fields] OR "assayed"[All Fields] OR "assaying"[All Fields] OR "assays"[All Fields] OR ("analysis"[MeSH Subheading] OR "analysis"[All Fields] OR "assay"[All Fields] OR "biological assay"[MeSH Terms] OR ("biological"[All Fields] AND "assay"[All Fields]) OR "biological assay"[All Fields] OR "assay s"[All Fields] OR "assayed"[All Fields] OR "assaying"[All Fields] OR "assays"[All Fields]) OR ("biomarker s"[All Fields] OR "biomarkers"[MeSH Terms] OR "biomarkers"[All Fields] OR "biomarker"[All Fields]) OR ("biomarker s"[All Fields] OR "biomarkers"[MeSH Terms] OR "biomarkers"[All Fields] OR "biomarker"[All Fields]) OR "bio-signature"[All Fields] OR "bio-signatures"[All Fields] OR ("express"[All Fields] OR "expresse"[All Fields] OR "expresses"[All Fields] OR "expressing"[All Fields] OR "expressions"[All Fields] OR "gene expression"[MeSH Terms] OR ("gene"[All Fields] AND "expression"[All Fields]) OR "gene expression"[All Fields] OR "expressed"[All Fields] OR "expression"[All Fields]) OR ("marker"[All Fields] OR "markers"[All Fields]) OR ("marker"[All Fields] OR "markers"[All Fields]) OR ("profile"[All Fields] OR "profiled"[All Fields] OR "profiler"[All Fields] OR "profilers"[All Fields] OR "profiles"[All Fields] OR "profiling"[All Fields] OR "profilings"[All Fields]) OR ("profile"[All Fields] OR "profiled"[All Fields] OR "profiler"[All Fields] OR "profilers"[All Fields] OR "profiles"[All Fields] OR "profiling"[All Fields] OR "profilings"[All Fields]) OR ("profile"[All Fields] OR "profiled"[All Fields] OR "profiler"[All Fields] OR "profilers"[All Fields] OR "profiles"[All Fields] OR "profiling"[All Fields] OR "profilings"[All Fields]) OR ("protein domains"[MeSH Terms] OR ("protein"[All Fields] AND "domains"[All Fields]) OR "protein domains"[All Fields] OR "signature"[All Fields] OR "signatures"[All Fields]) OR ("protein domains"[MeSH Terms] OR ("protein"[All Fields] AND "domains"[All Fields]) OR "protein domains"[All Fields] OR "signature"[All Fields] OR "signatures"[All Fields]) OR ("biomarkers"[MeSH Terms] OR "biomarkers"[All Fields] OR ("surrogate"[All Fields] AND "endpoint"[All Fields]) OR "surrogate endpoint"[All Fields]) OR ("research design"[MeSH Terms] OR ("research"[All Fields] AND "design"[All Fields]) OR "research design"[All Fields] OR "test"[All Fields]) OR ("test s"[All Fields] OR "tested"[All Fields] OR "testing"[All Fields] OR "testings"[All Fields] OR "tests"[All Fields]) OR "tool"[All Fields] OR ("tool s"[All Fields] OR "tools"[All Fields])) AND ("blood"[All Fields] OR "SERUM"[All Fields] OR "plasma"[All Fields]) AND ("Tuberculosis"[All Fields] OR "TB"[All Fields]) AND ((("hiv"[MeSH Terms] OR "hiv"[All Fields] OR ("human"[All Fields] AND "immunodeficiency"[All Fields] AND "virus"[All Fields]) OR "human immunodeficiency virus"[All Fields]) AND "OR"[All Fields]) AND ("hiv"[MeSH Terms] OR "hiv"[All Fields]))

Embase **07 November 2023**

| 1. | (Tuberculosis or TB).mp. [mp=title, abstract, heading word, drug trade name, original title, device manufacturer, drug manufacturer, device trade name, keyword heading word, floating subheading word, candidate term word] |  |  |  |  |
| --- | --- | --- | --- | --- | --- |
| 2. | exp "Africa south of the Sahara"/ |  |  |  |  |
| 3. | exp Human immunodeficiency virus/ |  |  |  |  |
| 4. | ("assay" or "assays" or "biomarker" or "biomarkers" or "bio-signature" or "bio-signatures" or "expression" or "marker" or "markers" or "profile" or "profiling" or "profiles" or "signature" or "signatures" or "surrogate endpoint" or "test" or "tests" or "tool" or "tools").mp. [mp=title, abstract, heading word, drug trade name, original title, device manufacturer, drug manufacturer, device trade name, keyword heading word, floating subheading word, candidate term word] |  |  |  |  |
| 5. | ("blood" or "SERUM" or "plasma").mp. [mp=title, abstract, heading word, drug trade name, original title, device manufacturer, drug manufacturer, device trade name, keyword heading word, floating subheading word, candidate term word] |  |  |  |  |
| 6. | 1 and 2 and 3 and 4 and 5 |  |  |  |  |
